# The experiences and opinions of Polish medical personnel regarding limitations of futile treatment in intensive care units – A questionnaire study

**DOI:** 10.1017/S1478951525101399

**Published:** 2025-12-29

**Authors:** Paweł Melchior Pasieka, Wojciech Skupnik, Magdalena Fronczek, Roman Jaeschke, Wojciech Szczeklik

**Affiliations:** 1 Centre for Intensive Care and Perioperative Medicine, Jagiellonian University Medical College, Kraków, Poland; 2 Department of Medicine, McMaster University, Hamilton, ON, Canada; 3 Department of Health Research Methods, Evidence, and Impact, McMaster University, Hamilton, ON, Canada

**Keywords:** Futile treatment, intensive care, questionnaire study, end-of-life care, personnel

## Abstract

**Objectives:**

Futile treatment is defined as maintenance of organ function without achieving meaningful goals of care. Poland is characterized by low prevalence of introducing limitations of treatment in intensive care units (ICUs). The aim of the study was to conduct a questionnaire study to evaluate the approach of Polish medical personnel to futile treatment in the ICUs.

**Materials and Methods:**

We conducted an anonymous questionnaire study during a national intensive care conference in April 2023. We collected data on participants’ experiences with limiting futile treatment and their demographics. Statistical analysis comparing the responses between respondents with shorter (less than 10 years) or longer (10 years or more) work experience was conducted with a chi-squared test with residual analysis and Bonferroni correction.

**Results:**

354 respondents completed the questionnaire. Most participants (94.5%) found discussing end-of-life care with patients important. Additionally, 81.6% believed that the medical personnel should be more decisive than the patient’s family regarding end-of-life care decisions. While 81% were aware of the existence of futile treatment protocol, only 35% used it regularly. Fear of legal consequences (61.9%) or family’s reaction (55.6%) were the most common reasons for not adhering to existing guidelines. Improving hospital procedures (83.6%) and proper legislation (67.2%) were commonly suggested measures to improve end-of-life care. Respondents with shorter work experience more often reported no awareness of futile treatment protocol (28.7% vs. 6.9%, *p* < 0.001) as well as no experience discussing treatment limitations with patients (24.6% vs. 8.2%, *p* < 0.001) or their families (20.0% vs. 3.8%) compared to the clinicians with longer work experience.

**Significance of results:**

Despite widespread recognition of the unethical nature of futile treatment, it remains controversial among Polish ICU clinicians. Improvement of legislation and hospital procedures could contribute to improvement of clinicians’ and patients’ well-being when facing end-of-life care decisions.

## Introduction

According to Polish guidelines futile therapy refers to medical interventions that artificially maintain organ function in patients without a realistic prospect of achieving goals of care. Such practice prolongs patients’ suffering without any benefit and is widely considered a medical and ethical error (Kübler et al. 2014). However, Poland is characterized by particularly low incidence of life-sustaining treatment limitations compared to other European countries (Guidet et al. 2020; Wernly et al. 2022). The precise opinions and sentiments of Polish intensive care clinicians regarding end-of-life and futile care remain unknown.

The main aim of the study was to conduct the questionnaire study to evaluate the approach of Polish medical personnel to the problem of futile treatment in the ICUs.

## Materials and methods

The electronic questionnaire was administered to participants of the intensive care conference ‘Forum Intensywnej Terapii’ held in April 2023. The congress gathered clinicians practicing intensive care across the whole country. Roughly 700 participants participated in the conference. The questionnaire consisted of 10 questions regarding goals of care, communication between physicians and patients or patients’ families, futile treatment protocol, as well as baseline data (country, region, gender, specialty, work experience, type of hospital).

The results were collected anonymously. The statistical analysis was conducted with SPSS version 29. The chi-squared test was conducted when comparing the responses between respondents with shorter (<10 years) and longer (>10 years) work experience. *P*-value of 0.05 was considered a threshold for statistical significance. In post-hoc assessments, residual analysis was used with Bonferroni correction for multiple comparison adjustment.

## Results

Overall, 354 questionnaires were collected, corresponding to a response rate of approximately 50%. The detailed breakdown of participants’ characteristics is summarized in Table 1. We present questions asked in Table 2.

**Table 1.** Demographic data of patients

Region (Voivodeship)	
*Lower Silesian Voiv.*	21 (5.9%)
*Kuyavian-Pomeranian Voiv.*	30 (8.5%)
*Łódź Voiv.*	15 (4.2%)
*Lublin Voiv.*	7 (2.0%)
*Lubuskie Voiv.*	5 (1.4%)
*Lesser Poland Voiv.*	69 (19.5%)
*Mazovia Voiv.*	64 (18.1%)
*Opole Voiv.*	8 (2.3%)
*Subcarpathian Voiv.*	22 (6.2%)
*Podlaskie Voiv,*	11 (3.1%)
*Pomeranian Voiv.*	25 (7.1%)
*Silesian Voiv.*	37 (10.5%)
*Holy Cross Voiv.*	5 (1.4%)
*Warmian-Masurian Voiv.*	9 (2.5%)
*Greater Poland Voiv.*	12 (3.4%)
*West Pomeranian Voiv.*	14 (4.0%)
**Sex**	
*Female*	216 (61.0%)
*Male*	138 (39.0%)
**Occupation**	
*Physician, specialist*	159 (44.9%)
*Physician, intern/residency*	79 (22.3%)
*Nurse*	83 (23.4%)
*Paramedic*	19 (5.4%)
*Students*	13 (3.7%)
**Years of work**	
*Less than 10 years of work*	195 (55.1%)
*10 years of work or more*	159 (44.9%)
**Place of work**	
University hospital	114 (32.2%)
Other hospital	240 (67.8%)

**Table 2.** Questions asked in the questionnaire study

**Question 1. In your opinion, should we talk to patients about end-of-life care and futile treatment?**
*Yes*
*No*
**Question 2. Assuming that the diagnosis is known, when is the optimal time to discuss goals of care with patients?**
*Immediately after admission to the hospital or in first hours/days of hospitalization*
*After significant deterioration of patient’s general condition*
*After ICU admission*
*It should never be discussed with patients*
**Question 3. What should be the balance of decision-making authority regarding treatment limitations between family and medical personnel?**
*Definitely more balanced toward the personnel*
*Slightly more balanced toward personnel*
*Equally balanced*
*Slightly more balanced toward the family*
*Definitely more balanced toward the family*
**Question 4. Do you find it difficult to discuss futile treatment with the patient, even if you consider it important?**
*Definitely yes*
*Rather yes*
*Rather no*
*Definitely no*
*I don’t know/I don’t discuss such issues with patients*
**Question 5. Do you find it difficult to discuss futile treatment with the patient’s family, even if you consider it important?**
*Definitely yes*
*Rather yes*
*Rather no*
*Definitely no*
*I don’t know/I don’t discuss such issues with families*
**Question 6. Are you familiar with the protocol and guidelines on limiting futile treatment, introduced in 2014 by prof. Kubler’s team and recommended by the Polish Society of Anesthesiology and Intensive Care?**
*Yes and I use it regularly*
*Yes and I use it rarely*
*Yes and I never use it*
*No*
**Question 7. What could be the possible reasons for not adhering to the guidelines and futile treatment protocol?^1^**
*Lack of usefulness*
*The protocol formula*
*Time restraints*
*Fear of legal consequences*
*Fear of family’s reaction*
*Religious objections*
**Question 8. In your opinion, which initiatives could expand the clinicans’ knowledge about the futile treatment?^1^**
*Exercise in medical school*
*Internal courses in the ICU*
*Interdisciplinary courses in the hospital*
*Introducing protocols as standard procedures in hospital*
*Discussing the subject on the conferences*
*Introducing the protocol into the legislation*
**Question 9. Have you talked more often to patients and/or their families about futile therapy since the beginning of the COVID-19 pandemic in Poland (March 2020)?**
*Yes, definitely more often*
*Yes, somewhat more often*
*No, I use it with the same frequence*
*No, I use it more rarely*
*I don’t know/I have never talked that subject*
**Question 10. Have you used the futile treatment protocol more frequently since the beginning of the COVID-19 pandemic in Poland (March 2020)?**
*Yes, definitely more often*
*Yes, somewhat more often*
*No, I use it with the same frequence*
*No, I use it more rarely*
*I don’t know/I have never talked that subject*

1 Multiple choice possible

***Question 1****. In your opinion, should we talk to patients about end-of-life care and futile treatment?*

The vast majority (98.5% of respondents) stated that talking to patients about goals of care/futile treatment is important in practice, with less than 2% thinking otherwise (Table 3).

**Table 3.** Responses to Question 1: *In your opinion, should we talk to patients about end-of-life care and futile treatment?*

	Overall	Work experience <10 years	Work experience ≥10 years	*p*
*Definitely yes*	329 (92.9%)	179 (91.8%)	150 (94.3%)	0.314
*Rather yes*	20 (5.6%)	14 (7.2%)	6 (3.8%)	
*I don’t know/I have no opinion*	6 (1.4%)	2 (1.1%)	4 (2.5%)	
*Definitely no*	5 (1.4%)	2 (0.5%)	3 (1.9%)

***Question 2****. Assuming that the diagnosis is known, when is the optimal time to discuss goals of care with patients?*

About 70.3% of respondents believed that patients should be talked to about goals of care immediately after hospital admission, with further 27.1% pointing to significant deterioration of patient’s condition and only 2.5% indicated such discussion should happen only after ICU admission (Table 4).

**Table 4.** Responses to Question 2: *Assuming that the diagnosis is known, when is the optimal time to discuss goals of care with patients?*

	Overall	Work experience <10 years	Work experience ≥10 years	*p*-Value
Within first hours of admission	249 (70.3%)	135 (69.2%)	114 (71.7%)	0.876
After significant deterioration of patient’s condition	96 (27.1%)	55 (28.2%)	41 (25.8%)	
After ICU admission	9 (2.5%)	5 (2.6%)	4 (2.5%)	
It should never be discussed with patient	0 (0%)	0 (0%)	0 (0%)

***Question 3.*** *What should be the balance of decision-making authority regarding treatment limitations between family and medical personnel?*

About 81.6% of respondents believed that medical personnel should have more influence than family members when undertaking end-of-life care decisions (Table 5).

**Table 5.** Responses to Question 3: *What should be the balance of decision-making authority regarding treatment limitations between family and medical personnel?*

	Overall	Work experience <10 years	Work experience ≥10 years	*p*-Value
*Definitely more balanced toward the personnel*	160 (45.2%)	78 (40%)	82 (51.6%)	0.109
*Slightly more balanced toward the personnel*	129 (36.4%)	78 (40%)	51 (32.1%)	
*Equally balanced*	58 (6.4%)	34 (17.4%)	24 (15.1%)	
*Slightly more balanced toward the family*	6 (1.7%)	5 (2.6%)	1 (0.6%)	
*Definitely more balanced toward the family*	1 (0.3%)	0 (0%)	1 (0.6%)

***Question 4.*** *Do you find it difficult to discuss futile treatment with the patient, even if you consider it important?*

***Question 5.*** *Do you find it difficult to discuss futile treatment with the patient’s family, even if you consider it important?*

51.2% of participants reported discomfort when discussing end-of-life care topics with patients while 48.8% had difficulty talking to their families, Respondents with longer work experience significantly more frequently reported having no difficulties (Tables 6 and 7].

**Table 6.** Responses to Question 4: *Do you find it difficult to discuss futile treatment with the patient, even if you consider it important?*

	Overall	Work experience <10 years	Work experience ≥10 years	*p*-Value
*Definitely yes*	65 (18.4%)	40 (20.5%)	25 (15.7%)	**<0.001**
*Rather yes*	116 (32.8%)	64 (32.8%)	52 (32.7%)	
***I don’t know/I don’t discuss such issues with patients*^*a*^**	**61 (17.2%)**	**48 (24.6%)**	**13 (8.2%)**	
*Rather no*	89 (25.1%)	40 (20.5%)	49 (30.8%)	
***Definitely no*^*a*^**	**23 (6.5%)**	**3 (1.5%)**	**20 (12.5%)**	

a Significant differences found in residual analysis.

**Table 7.** Responses to Question 5: *Do you find it difficult to discuss futile treatment with the patient’s family, even if you consider it important?*

	Overall	Work experience <10 years	Work experience ≥10 years	*p*-Value
*Definitely yes*	60 (16.9%)	41 (21.0%)	19 (11.9%)	**<0.001**
*Rather yes*	113 (31.9%)	64 (32.8%)	49 (30.8%)	
***I don’t know/I don’t discuss such issues with patients*^*a*^**	**45 (12.7%)**	**39 (20.0%)**	**6 (3.8%)**	
***Rather no*^*a*^**	**102 (12.9%)**	**40 (20.5%)**	**62 (39.0%)**	
*Definitely no*	34 (4.3%)	11 (5.6%)	23 (14.5%)	

a Significant differences found in residual analysis.

***Question 6.*** *Are you familiar with the protocol and guidelines on limiting futile treatment, introduced in 2014 by prof. Kubler’s team and recommended by the Polish Society of Anesthesiology and Intensive Care?*

81.2% were aware of futile treatment protocol, however only 35.3% used it regularly. Residual analysis revealed that respondents with shorter work experience significantly more often admitted not knowing about the protocol (Table 8).

**Table 8.** Responses to Question 6*: Are you familiar with the protocol and guidelines on limiting futile treatment, introduced in 2014 by prof. Kubler’s team and recommended by the Polish Society of Anesthesiology and Intensive Care?*

	Overall	Work experience <10 years	Work experience ≥10 years	*p*-Value
***Yes, and I use it regularly*^*a*^**	**125 (35.3%)**	**50 (35.6%)**	**75 (47.2%)**	**<0.001**
*Yes, but I use it rarely*	92 (26.0%)	54 (27.7%)	38 (23.9%)	
***Yes, but I never use it* ^*a*^**	**70 (19.8%)**	**35 (17.9%)**	**35 (22.0%)**	
***No*^*a*^**	**67 (18.9%)**	**56 (28.7%)**	**11 (6.9%)**	

a Significant differences found in residual analysis.

***Question 7.*** *What could be the reasons for not adhering to the guidelines and the futile treatment protocol?* (multiple choice question)

Fear of legal consequences (61.9%), fear of family reactions (55.6%) and lack of proper knowledge (39.3%) were the leading causes for protocol implementation hesitancy. The participants with shorter work experience were significantly more likely to point to lack of knowledge and time constraints (Table 9).

**Table 9.** Responses to Question 7: *What could be the reasons for not adhering to the guidelines and the futile treatment protocol?*

	Overall	Work experience <10 years	Work experience ≥10 years	*p*-Value
*Lack of usefulness*	2 (1.1%)	2 (1%)	0 (0%)	0.20
***Lack of knowledge***	**139 (39.3%)**	**99 (50.8%)**	**40 (25.2%)**	**<0.001**
***Time constraints***	**80 (22.6%)**	**54 (27.7%)**	**26 (16.4%)**	**0.011**
*Fear of legal consequences*	219 (61.9%)	117 (60.0%)	102 (64.2%)	0.424
*Fear of family reaction*	197 (55.6%)	111 (56.9%)	86 (54.1%)	0.593
*Religious objections*	43 (12.1%)	29 (14.9%)	14 (8.8%	0.082

***Question 8.*** *In your opinion, which initiatives could expand clinicians’ knowledge about futile treatment?* (multiple choice question)

Implementation of the futile therapy protocol as a standard hospital procedure and its legal recognition were most frequently indicated as possible means of increasing the physicians’ knowledge of the existing protocol. Respondents with shorter work experience more frequently pointed to usefulness of internal courses in the ICU and discussions at medical conferences (Table 10).

**Table 10.** Responses to Question 8: *In your opinion, which initiatives could expand clinicians’ knowledge about futile treatment?*

	Overall	Work experience <10 years	Work experience ≥10 years	*p*-Value
*Courses in medical school*	181 (51.1%)	106 (54.4%)	75 (47.2%)	0.178
***Internal courses in the ICU***	**166 (46.9%)**	**101 (51.8%)**	**65 (40.9%)**	**0.041**
*Interdisciplinary courses in the hospital*	229 (64.7%)	124 (63.6%)	105 (66.0%)	0.632
*Implementation of protocols as standard procedures in the hospital*	296 (83.6%)	167 (85.6%)	129 (81.1%)	0.254
***Discussing the topic on medical conferences***	**238 (67.2%)**	**141 (72.3%)**	**97 (61.0%)**	**0.024**
*Incorporation of the protocol into legislation*	238 (67.2%)	296 (67.7%)	238 (67.2%)	0.939

***Question 9*.** *Have you talked more often to patients and/or their families about futile therapy since the beginning of the COVID-19 pandemic in Poland (March 2020)?*

***Question 10.*** *Have you used the futile treatment protocol more frequently since the beginning of the COVID-19 pandemic in Poland (March 2020)?*

Most respondents reported no change in the frequency of discussing goals of care or implementing protocol after COVID-19 pandemic; however, roughly 30% admitted to doing so somewhat or significantly more often [Table 11; Table 12].

**Table 11.** Responses to Question 9: *Have you talked more often to patients and/or their families about futile therapy since the beginning of the COVID-19 pandemic in Poland (March 2020)?*

	Overall	Work experience <10 years	Work experience ≥10 years	*p*-Value
**I don’t know/I have never discussed that subject^a^**	**81 (22.9%)**	**66 (33.8%)**	**15 (9.4%)**	**<0.001**
No, definitely less often	4 (1.1%)	2 (1.0%)	2 (1.3%)	
No, somewhat less often	4 (1.1%)	3 (1.5%)	1 (0.5%)	
**No, with the same frequence^a^**	**146 (41.2%)**	**60 (30.6%)**	**86 (54.1%)**	
Yes, somewhat more often	53 (15.0%)	28 (14.4%)	25 (15.7%)	
Yes, definitely more often	66 (18.6%)	36 (18.5%)	30 (18.9%)	

a Statistically significant difference found in residual analysis.

**Table 12.** Responses to Question 10: Have you used the futile treatment protocol more frequently since the beginning of the COVID-19 pandemic in Poland (March 2020)?

	Overall	Work experience <10 years	Work experience ≥10 years	*p*-Value
**I don’t know/I have never talked that subject^a^**	**117 (22.9%)**	**84 (43.1%)**	**33 (20.8%)**	**<0.001**
No, definitely less often	4 (1.1%)	1 (0.5%)	3 (1.9%)	
No, somewhat less often	1 (0.3%)	0 (0%)	1 (0.6%)	
**No, with the same frequence^a^**	**129 (36.4%)**	**55 (28.2%)**	**74 (46.5%)**	
Yes, somewhat more often	53 (15.0%)	22 (11.3%)	21 (13.2%)	
Yes, definitely more often	66 (18.6%)	33 (16.9%)	27 (17.0%)	

^a^ *Statistically significant difference found in residual analysis.*

## Discussion

The topic of futile treatment is a contentious issue that has recently gained increasing recognition among healthcare professionals. According to the literature, approximately 80–90% of ICU physicians have already practiced withholding or withdrawing therapy (Cardoso et al. 2003; Giannini et al. 2003; Kübler et al. 2011). Our respondents widely acknowledged the importance of discussing futile treatment with patients and their relatives.

However, many clinicians admit administering or witnessing futile treatment for a variety of reasons (Huynh et al. 2013). Several guidelines regarding adequate end-of-life care in ICUs have been introduced to support physicians facing ethical dilemmas (Kesecioglu et al. 2024). Polish guidelines recommend implementing a protocol for documenting decisions on withholding or withdrawing life-sustaining therapies, while ensuring the continuation of palliative and comfort care. (Kübler et al. 2014).

Recently, considerable attention has been devoted in the Polish medical community to the issue of perceived overzealous treatment, futile care or end-of-life care. These terms are sometimes (and potentially inappropriately) used interchangeably. The COVID-19 pandemic has caused renewed discussion regarding these phenomena as well as the implications of limited ICU resources (Zadi et al. 2024). Poland participated in both Very Old Intensive Patients studies, which addressed limitations of treatment (Flaatten et al. 2017; Guidet et al. 2020). Several national conferences held panels dedicated to the topic of futile treatment. This issue has been also explored by experts in other populations, including pediatric ICU patients (Bartkowska-Śniatkowska et al. 2021) and patients in internal medicine wards (Szczeklik et al. 2023). The recent novelization of the Polish Code of Medical Ethics introduced a new paragraph specifically prohibiting futile treatment (Alichniewicz and Alichniewicz 2024).

Despite those efforts, there is still much to be learned and done. Several studies identified Poland as a country with comparatively low prevalence of treatment limitations (including withdrawal and withholding). In a multicenter study of older patients in the European ICUs, 19.8% older patients in Poland were subjected to some form of treatment limitation compared to 34.4% in the overall European sample (Guidet et al. 2020). Polish clinicians limit life-sustaining treatment less frequently than their Scandinavian counterparts (Siewiera et al. 2019). Our survey supports this observation: although most clinicians were aware of the protocol and recognized its importance, they admitted to never or only rarely using it.

Our study attempted to identify barriers to implementing the existing professional protocol. The most frequently reported obstacle was the fear of legal consequences. In a survey of Polish nurses, the fear of legal consequences was also a leading reason to continue what was perceived as futile therapy. (Damps et al. 2022). This sentiment is understandable, as futile treatment is virtually absent from the Polish legal framework. Although numerous legal experts believe that the Polish law does support limitations of futile treatment, this is never explicitly stated in the Polish legal documents (Szeroczyńska 2013). Moreover, Poland lacks a ‘no-fault’ rule, and a physician suspected of medical error can be prosecuted based on the Penal Code (Bargiel and Sułkowski 2023). Introducing an act of law dedicated to the issue, similarly to the German and French solutions (Graw et al. 2012; Lesieur et al. 2015), might significantly improve clinicians’ confidence when approaching end-of-life care ethical problems, especially the issue of futile treatment. The impact of legal frameworks on ethical decision-making in ICUs has been emphasized in European guidelines on limitations of treatment in the ICUs (Kesecioglu et al. 2024).

Another major factor highlighted by respondents was fear of the patient’s family objections. This is less modifiable but aligns with findings from other surveys. More than half of Polish nurses reported fear of being accused of malpractice by a patient’s family as a reason for continuing futile treatment (Damps et al. 2022). It is worth noting that, in addition to proper legislation and hospital procedures, effective communication with family along with psychosocial and spiritual support, contributes to better cooperation and mutual understanding between the health care team and relatives during end-of-life discussions (Woźnica-Niesobska et al. 2020). One study found out that prognostic communication, documentation of DNR orders and limitations of treatment, and social worker support positively influenced the deceased patients’ relatives’ satisfaction with ICU care (Chou et al. 2022). At the same time, it is generally acknowledged that physicians are not obliged to follow relatives’ wishes in every case by prolonging treatment deemed futile and unethical (Szeroczyńska 2013). Such situations, however, may lead to conflicts arising from differing perceptions of what constitutes futility.

In our study, respondents with less work experience reported feeling less comfortable discussing end-of-life care with patients and their relatives and indicated that a lack of knowledge may underlie the non-implementation of the futile treatment protocol. This association between lower professional experience and greater difficulty in addressing futility is consistent with an American study, which found that intensive care fellows differed significantly from more experienced clinicians in their assessment of medical futility (Neville et al. 2015). Simultaneously a questionnaire study of Polish medical students found that even though most Polish students heard about futile treatment, only 39% could define it properly (Pełczyński et al. 2023). These findings along with our study point out the necessity of putting more focus on the topic of futile treatment in Polish medical education.

### Limitations

Our study has some limitations. The questionnaires were filled by voluntary respondents, which might increase the risk of omitting physicians who were not familiar with the concept of futile therapy or unwilling to state their opinion. Due to our questionnaire distribution method, it was not possible to calculate the exact response rate.

## Conclusions

Even though there is significant awareness among Polish clinicians regarding futile treatment and end-of-life care/protocol in ICUs, there is still a significant room for improvement regarding knowledge of and implementation of guidelines for limitations of treatment in situations deemed futile, especially among clinicians with shorter work experience. Our study underlines the need for optimizing legal and formal frameworks allowing open and transparent discussion about different aspects of end-of-life care.
